# Evaluating geostatistical modeling of exceedance probability as the first step in disease cluster investigations: very low birth weights near toxic Texas sites

**DOI:** 10.1186/1476-069X-13-47

**Published:** 2014-06-07

**Authors:** James A Thompson, Wesley T Bissett, Anne M Sweeney

**Affiliations:** 1 Department of Large Animal Clinical Sciences, College of Veterinary Medicine and Biomedical Sciences, Texas A&M University, College Station, TX 77843-4475, USA; 2 Department of Epidemiology and Biostatistics, School of Rural Public Health, Texas A&M Health Science Center, College Station, College Station, TX 77843-1266, USA

## Abstract

**Background:**

The first step in evaluating potential geographic clusters of disease calls for an evaluation of the disease risk comparing the risk in a defined location to the risk in neighboring locations. Environmental exposures, however, represent continuous exposure levels across space not an exposure with a distinct boundary. The objectives of the current study were to adapt, apply and evaluate a geostatistical approach for identifying disease clusters.

**Methods:**

The exceedance probability for very low birth weight (VLBW; < 1.5 kg) infants was mapped using an Intrinsic Conditional Autoregressive model. The data were applied to a 20 by 20 grid of 1 km^2^ pixels centered on each of the 13 National Priority List Superfund Sites in Harris County, Texas.

**Results:**

Large clusters of VLBW were identified in close proximity to four of the 13 Superfund Sites. Three of the Superfund Sites, associated with disease clusters, were located close together in central Houston and these sites may have been surrounded by a single, confluent disease cluster.

**Conclusions:**

Geostatistical modeling of the exceedance probability for very low birth weights identified disease clusters of varying size, shape and statistical certainty near Superfund Sites in Harris County, Texas. The approach offers considerable potential as the first step for investigating potential disease clusters.

## Background

The National Center for Environmental Health (NCEH) defines a cluster as a greater-than-expected number of cases that occurs within a group of people in a geographic area over a defined period of time. The Centers for Disease Control and Prevention (CDC) provide guidelines for investigation of potential clusters but, in the United States, the investigation usually falls to the state health department. The first step, in the investigation, is usually a statistical evaluation of the likelihood of the disease distribution. Commonly the state health agency examines its health registry data and performs statistical testing comparing incidence rates among arbitrary geographic areas [1]. Once a significant statistical association is established, the latter steps are committed to identification of a cause for the disease cluster. However, most investigations end with the first step because of a failure to identify a critically small p-value [2,3].

Instead of investigating a risk boundary and estimating fixed risks on either side of the boundary, there could be advantages to evaluating a more continuous geographic risk using geostatistical modeling. By definition, geostatistical modeling collects data based on sampling coordinates then fits both a local risk estimate and a spatial covariance allowing for near neighbors to be more similar in risk. The estimated risks and spatial covariance enable the prediction of risk at un-sampled locations thus filling in the complete risk surface. When implemented using a fully Bayesian approach, the risk surface can be plotted using any estimate from the posterior, including the posterior probability that the relative risk (RR) estimate is greater than one [4]. This is often called the exceedance probability (EP). The EP is affected by both the magnitude and the precision of the relative risks which, in turn, are largely determined by the extent of spatial correlation. A geostatistical model of the EP could provide a powerful tool evaluating flexible cluster shapes and sizes and could estimate the risk and the risk uncertainty for those living near the site.

Abnormal birth conditions are among the most frequently investigated health conditions for suspected geographic clustering. Fetuses are known to be especially susceptible to environmental toxins and the public often perceives clusters of preterm births and frequently blames local pollution. Very low birth weights (VLBW; birth weight < 1500 g) and very preterm delivery (VPTD; gestational periods of less than 32 weeks) are common conditions that have been studied extensively. The two conditions contribute markedly to neonatal morbidity and mortality and lifelong health care costs [5]. The two conditions are very highly correlated but many consider VPTD as the outcome of more biologic interest [6]. However, VLBW has often been studied as a surrogate for VPTD because of how objectively birth weight is measured relative to the estimation of gestational length [7].

The objectives of this study were to adapt, apply and evaluate geostatistical modeling of exceedance probability for VLBW near specific toxic sites, in Harris County, Texas. Harris County, located within the Houston–The Woodlands–Sugar Land metropolitan area, is the third most populous county in the United States and has 13 National Priority List (NPL) Superfund Sites, the sixth most of any county in the nation. Harris County has a diverse population, including large groups of disadvantaged residents that live near toxic sites and have high birth rates.

## Methods

The use of protected health information from the birth certificate database was approved by two Institutional Review Boards, the Texas Department of State Health Services (TDSHS) and Texas A&M University. Birth records, including health related fields, were retrieved from TDSHS for the period January 1, 1990 to December 31, 2002. Geocoding was performed by the TDSHS, based on street addresses, and was 87 percent complete. The latitude and longitude of birth locations were projected into Universal Transverse Mercator 1983 (UTM83), Zone 14 units. All births were located using the mothers address at birth and rounding the geocoordinates to the nearest 1 km unit.

The identities and locations of the 13 National Priority List Superfund Sites were first identified using latitude and longitude given on the Texas Site Status Summaries on the EPA Program Region 6 Superfund website (http://www.epa.gov/region6/6sf/6sf-tx.htm). The Superfund sites were then visually identified on satellite imagery and the apparent centroid was used as the location.

The Intrinsic Conditional Autoregressive (ICAR) model was used to model the relative risk (RR), defined as the number of cases divided by the number of expected cases [8]. Besag et al. [8] referred to the smallest geographical areas as pixels and we adopt that terminology. The UTM83 coordinate system was used with a distance scale of 1 km between coordinates and modeled the 20 by 20 grid surrounding each of the toxic sites. Each x,y coordinate represented the centroid of a 1 km x 1 km pixel. For each of the i = 400 pixels, the number of cases (*Y*_*i*_) was counted and the expected number of cases (*E*_*i*_) was the product of the number of births, over the 12-year study period and the overall expected morbidity ratio for VLBW. In some locations *E*_*i*_ was zero because there were zero births, thus creating a “structural” zero. To accommodate the structural zeros, a zero-inflated Poisson, using a two group mixture model recommended by Lunn et al., [9] was adapted as follows: For *i* = 400 spatial locations and for *j* = 2 groups,

$$Y_{i}∼\mathrm{Poisson}\;\left(m_{i}\right)$$

Where

$$m_{i=}\mathrm{grou}p_{j}*mu_{i}$$

and

$$\mathrm{grou}p_{j}\sim \mathrm{Bernoulli}\left(p_{j}\right)$$

Where *p* takes a value of 0 when no births were located within the pixel and a value of 1 when 1 or more births were observed within the pixel. The log(*mu*_*i*_) was then modeled linearly as a function of the expected rate of VLBW, an intercept (α) and Spatial term (*S*_*i*_).

$$\mathrm{Log}\;\left(mu_{i}\right)=\mathrm{Log}\;\left(E_{i}\right)+\alpha +S_{i}$$

All models employed Bayesian inference, with vague or flexible prior beliefs and a Markov Chain Monte Carlo (MCMC) implementation. The MCMC implementation was performed by use of WinBUGS version 1.4.3 [10]. The initial 5,000 iterations were discarded to allow for convergence and every hundredth of the following 500,000 iterations were sampled for the posterior distribution. Observing convergence of two chains with widely different initial values checked convergence to the posterior distribution.

The parameterization used for Geographical Information System (GIS) evaluation was the exceedance probability defined as the likelihood that the spatial RR estimate was greater than one and was taken directly from the full posterior distributions of the relative risk estimates. Initial GIS evaluation was performed on GeoBUGS version 1.2 and further GIS analysis was performed using ArcMap 10.

Three hierarchical priors were used and the results compared. The priors were gamma (0.5, 0.0005) for the variance; gamma (0.01, 0.01) for the variance and the uniform standard deviation prior (0, 100). The evaluation of these priors was performed in two steps. Maps of the risk surface for exceedance probability were compared using GIS and the full posteriors of the standard deviation of the spatial effects were also compared. Very low birth weight was the primary outcome of interest. For the purpose of sensitivity analyses, three other outcomes were evaluated: low birth weight (LBW; < 2500 g), preterm delivery (PTD; < 37 weeks gestation) and very preterm delivery (VPTD; < 32 weeks of gestation).

## Results

### VLBW

For the 12-year study period there were 777,553 births in Harris County, including 10,803 with VLBW (1.4%). The area of Harris County was 4410 pixels, for average birth rate and average VLBW rate of 176.3 and 2.4 per pixel, respectively.

### Cluster detection

**Figure 1:**
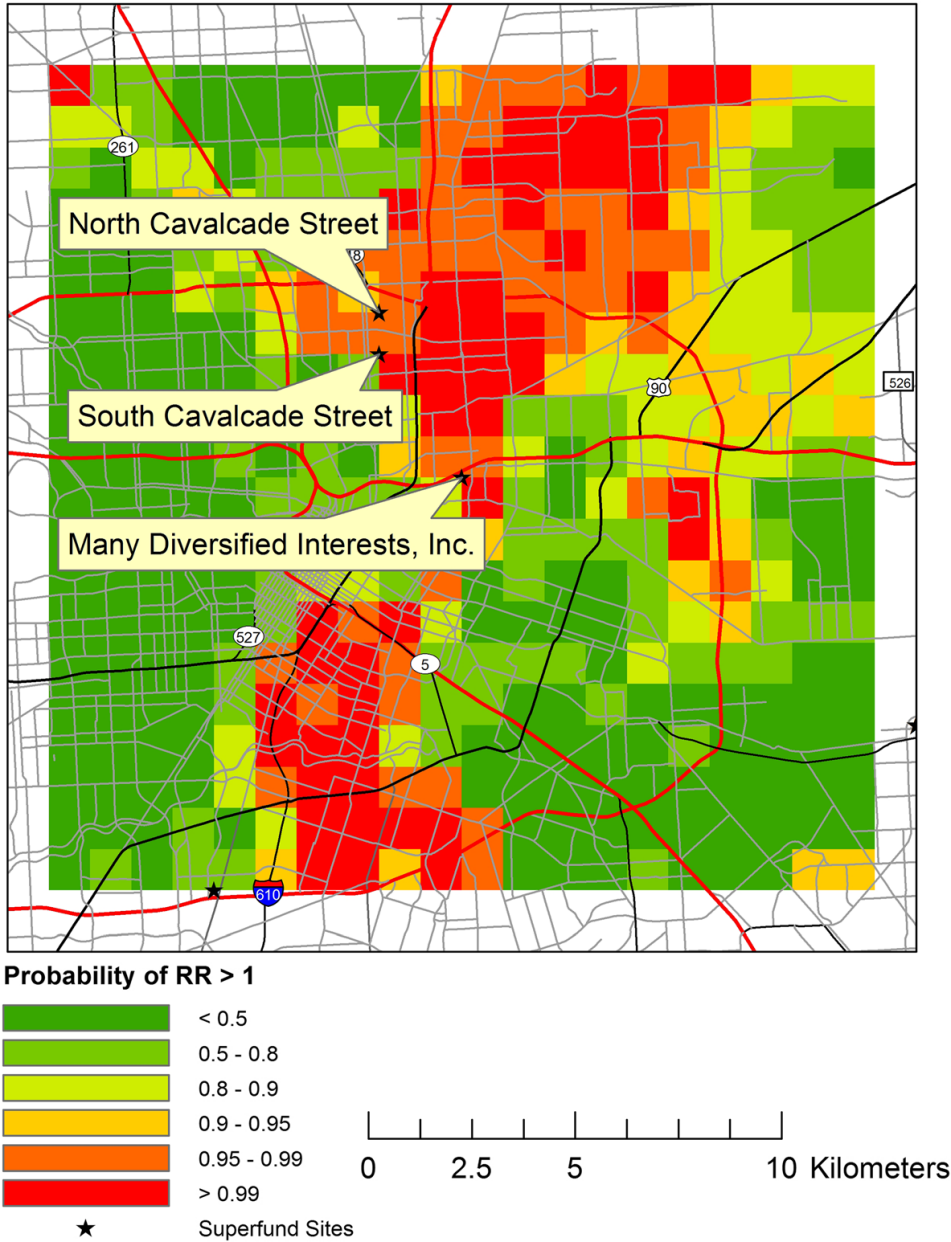
**Downtown Houston.** The exceedance probability for very low birth weights is plotted on the 20 km by 20 km grid centered at the Many Diversified Interests, Inc. Superfund Site but also including the North Cavalcade Street and South Cavalcade Superfund Sites.

**Figure 2:**
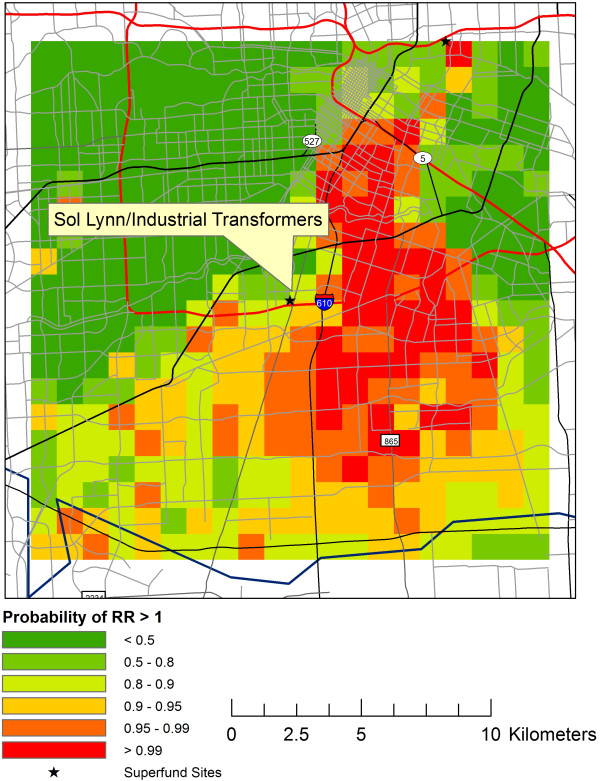
**Sol Lynn/Industrial transformers.** The exceedance probability for very low birth weights is plotted on the 20 km by 20 km grid centered on the Sol Lynn/Industrial Transformers Superfund Site.

Large clusters, with clusters defined as multiple adjacent pixels with EP > 99%, were identified within the study windows at six of the 13 Superfund Sites. Three of the sites were located close together in central Houston within the 610 Loop Interstate. These sites were the Many Diversified Interests, Inc., North Cavalcade Street and South Cavalcade Street Superfund Sites. The GIS layer centered at Many Diversified Interests, Inc. but also including the South Cavalcade Street and North Cavalcade Street Superfund Sites is presented in Figure 1. The Sol Lynn/Industrial Transformers Superfund Site had a large area of EP > 99%, extending to the east of the Superfund Site (Figure 2). Two other maps showed locations of very EP > 99% distal to the Superfund Sites, including 5-10 km southeast of the Crystal Chemical Co. Superfund Site and 5 km west of Geneva Industries/Fuhrman Energy Superfund Site. The latter cluster was confluent with the cluster detected near the Sol Lynn/Industrial Transformers Superfund Site.

### Standard deviation of neighborhood effects

The standard deviation (SD) among the pixels within a neighborhood is an essential parameter for modeling a risk gradient. The posterior for the SD was similar for the three Superfund Sites that were in close proximity in central Houston. For the seven locations without large clusters of high EP, the standard deviations were smaller, thus supporting more spatial homogeneity in the risks.

### Sensitivity analyses

The locations of large high EP clusters were very similar, for size, shape and location for LBW, PTD and VPTD. Sensitivity analyses, for the choice of priors, showed that the exceedance probabilities for VLBW were not sensitive to choice of priors.

## Discussion

The current standard is to compare disease rates using arbitrary boundaries like census tracts or political units when investigating potential disease clusters [1]. For example, a recent Endicott, NY investigation of low and very low birth weights and other birth disorders defined exposed and unexposed subjects using complex polygons defined by boundaries along city streets. The population on one side of the street was classified as exposed and the population on the other side as unexposed. The authors were investigating concern for Soil Vapor Intrusion following a large spill of toxic chlorinated hydrocarbons, trichloroethylene and perchloroethylene [11]. Soil Vapor Intrusion is a complex process in which contaminants move into the air from the soil creating an air-borne plume and exposure. This exposure process is certain to be continuous and not a process with a clear spatial boundary [12]. This type of exposure should also a concern for residents living near Texas Superfund sites. Other approaches to defining a cluster have been proposed including novel approaches that address flexibility in cluster shapes [13,14]. These approaches offer advantages over Kulldorff’s original spatial scan statistic [15] but still rely on a risk boundary. When the process is a continuous spatial one, the risks near the boundary but on opposite sides will be more alike than due to chance thus muting the risk estimate. For example, in the Endicott, NY study [11], it was unrealistic to model risks for subjects on opposite side of the street as belonging in either a full risk or no risk category. Models that are more continuous across space can estimate a gradient of risk and using the EP would offer the advantage of estimating and reporting the risk and risk certainty to those living near the investigated hazard.

Diggle and Ribeiro introduced Bayesian Kriging in which to covariance between sampled locations is estimated as a function of distance between all pairs of locations [16]. These linear geostatistical models have proven very useful in veterinary disease mapping because farms are not arranged in regular patterns and distances among the farm pairs can form the basis of the covariance [17-19]. Bayesian Kriging was a potential alternative for the current study but, the practical implementation using currently available computer resources is limited to rather small numbers of georeferenced locations. This limitation has been described by WinBUGS developers to be the result of the size of the covariance matrix [20]. The current study reduced the complexity of the spatial covariance matrix by using 1 km by 1 km pixels. The approach first projected the data from latitude and longitude to the UTM83 coordinate system. A coordinate system is needed because a degree of latitude consistently measures a distance of 111 km north to south but a degree of longitude measures a distance of 111 km east to west at the equator but 0 km at the north and south poles. In Houston, Texas a degree of longitude measures a distance of 97 km. The coordinate system is very important in estimating distance-based covariance but less important with ICAR modeling because correlation is identified among neighboring pixels without using distance measurements. The alternative of using latitude and longitude, in decimal degrees with geocoordinates rounded to the second decimal would produce very similar pixels that would measure approximately 1.10 km north to south and 0.97 km east to west when modeling central Texas health data. The projection and binning reduced the data complexity and as an additional advantage, protected individual patient identities. The criticism of the approach would include that the edges of the pixels were arbitrary and mothers located near but on opposite sides of the boundary would be more alike than by chance, similar to the criticism for large arbitrary locations. This concern is mitigated as the pixels become smaller and, also, the model specifically accounts for similarity of risk among neighboring pixels. Geostatistical modeling is intended for continuous space and the current “binning” procedure enabled a nearly continuous approach. The continued development of capacity for personal computing could render this adaptation unnecessary.

Spatial covariance should be considered dependent upon the existence of one or more spatially oriented causes. The search for local clusters, based on local spatial covariance, is considered both more useful and also more specifically addresses public concerns [21]. The current study deals with local spatial covariance by restricting the modeling of spatial covariance to locations very near the putative toxic source. The estimation of the spatial correlation is typically based on the data and an uninformative prior and the estimated spatial correlation determines the extent of spatial smoothing. In this context, spatial correlation refers to the similarity of map units that are being considered within the same “neighborhood.” This estimate can also be parameterized as the standard deviation (SD) or the variance of the units within a neighborhood. For consistency, we refer to the SD. The SD estimate can extend from zero, producing a map with the risk of each location being the same to a large standard deviation producing a mottled or more random map. Each study window consisted of 400 pixels. The spatial modeling considers each of the 400 pixels is as the center of a “neighborhood.” For pixels not on the boundary, the neighborhoods consist of nine, 1 km by 1 km pixels. The variation, for the SD estimate, among the 13 sites demonstrates the need to restrict the size of the study window when potential clusters are being evaluated because locations far from the cluster will have small SD estimates. In the current study, the seven study windows without clusters had a lower estimate for the SD which in turn produced a more uniform risk across the study window. The prior selected for the neighborhood effects also has the potential to obscure actual risk gradients. The current study compared three of the commonly recommended hierarchal variance priors. The gamma(epsilon, epsilon) prior was originally recommended in the WinBUGS manual as a precision prior [20]. The gamma (0.5, 0.0005) prior for the precision was recommended by Kelsall and Wakefield for situations in which the SD was poorly identifiable, the prior would not induce spatial structure by pulling the posterior of the SD away from zero [22]. This recommendation was in the context of larger map units and an objective of comparing risks among locations. In the current context of evaluating gradients of risk, it is important for the prior not to influence the posterior distribution of SD toward zero. Gelman promoted a variance prior that is uniform over a broad range on the standard deviation scale [23]. This prior is the most consistent with the prior beliefs of the investigators for the described approach to investigate risk gradients near a putative toxic source. The current study evaluated common conditions in a densely populated setting and the results were not sensitive to the prior selected. Future investigations should continue this sensitivity analysis. Much rarer diseases or very small spatial units, like those used in the current study may result in models with less identifiable standard deviations of neighborhood effects.

The first stage of cluster investigation is primarily statistical and evaluates the likelihood of a case excess. Once a case excess has been evaluated and the statistical certainty established, the objective becomes to identify the cluster cause. The identification of high risk locations does not provide sufficient evidence to implicate the suspected toxic source, but the results do support further investigation. Further investigation of potential causes should evaluate personal level covariates as causes ot the cluster. When personal attributes, like race, or personal exposure assessments can explain the geographic risk then the personal-level covariate should be considered the more specific cause of the cluster. The approach, presented here, can be extended by adjusting for personal-level covariates and then re-evaluating the GIS-based exceedance probabilities. This can be done without an ecologic bias. In contrast, cluster detection methods that model the risk as constant within a fixed cluster boundary are subject to an ecologic bias when the confounding between geographic risk and personal-level risks is assessed.

## Conclusions

Geostatistical modeling of the exceedance probability for very low birth weights identified disease clusters of varying size, shape and statistical certainty near Superfund Sites in Harris County, Texas. The approach offers potential as the first step for investigating potential disease clusters.

## Competing interests

The authors declare that they have no competing interests.

## Authors’ contributions

JAT carried out the modeling and GIS-evaluation and drafted the manuscript. WTB participated with the geographic modeling and GIS analysis and drafting the manuscript. AMS participated with epidemiologic advising and in drafting the manuscript. All authors read and approved the final manuscript.
